# Sheep Skin Odor Improves Trap Captures of Mosquito Vectors of Rift Valley Fever

**DOI:** 10.1371/journal.pntd.0001879

**Published:** 2012-11-01

**Authors:** David P. Tchouassi, Rosemary Sang, Catherine L. Sole, Armanda D. S. Bastos, Klaus Mithoefer, Baldwyn Torto

**Affiliations:** 1 International Centre of Insect Physiology and Ecology, Nairobi, Kenya; 2 Department of Zoology and Entomology, University of Pretoria, Pretoria, South Africa; 3 Centre for Virus Research, Kenya Medical Research Institute, Nairobi, Kenya; USAMRIID, United States of America

## Abstract

In recent years, the East African region has seen an increase in arboviral diseases transmitted by blood-feeding arthropods. Effective surveillance to monitor and reduce incidence of these infections requires the use of appropriate vector sampling tools. Here, trapped skin volatiles on fur from sheep, a known preferred host of mosquito vectors of Rift Valley fever virus (RVFV), were used with a standard CDC light trap to improve catches of mosquito vectors. We tested the standard CDC light trap alone (L), and baited with (a) CO_2_ (LC), (b) animal volatiles (LF), and (c) CO_2_ plus animal volatiles (LCF) in two highly endemic areas for RVF in Kenya (Marigat and Ijara districts) from March–June and September–December 2010. The incidence rate ratios (IRR) that mosquito species chose traps baited with treatments (LCF, LC and LF) instead of the control (L) were estimated. Marigat was dominated by secondary vectors and host-seeking mosquitoes were 3–4 times more likely to enter LC and LCF traps [IRR = 3.1 and IRR = 3.8 respectively] than the L only trap. The LCF trap captured a greater number of mosquitoes than the LC trap (IRR = 1.23) although the difference was not significant. Analogous results were observed at Ijara, where species were dominated by key primary and primary RVFV vectors, with 1.6-, 6.5-, and 8.5-fold increases in trap captures recorded in LF, LC and LCF baited traps respectively, relative to the control. These catches all differed significantly from those trapped in L only. Further, there was a significant increase in trap captures in LCF compared to LC (IRR = 1.63). Mosquito species composition and trap counts differed between the RVF sites. However, within each site, catches differed in abundance only and no species preferences were noted in the different baited-traps. Identifying the attractive components present in these natural odors should lead to development of an effective odor-bait trapping system for population density-monitoring and result in improved RVF surveillance especially during the inter-epidemic period.

## Author Summary

The East African region is a major epizootic center for endemic and emerging mosquito borne-arboviruses such as Rift Valley fever virus (RVFV), as evidenced by the increasing frequency and magnitude of this disease. The absence of vaccines or prophylactic drugs for most of these diseases emphasizes the need for accurate sampling of mosquito vector populations and testing for arboviruses. Accurate surveillance is crucial for early warning of potential or assessing mitigation of existing outbreaks. However, it is a challenge to sample mosquitoes in adequate numbers during the inter-epidemic periods (IEP) because this period is characterized by low mosquito population densities, sporadic transmission foci and low mosquito infection rates. Therefore more efficient tools are needed to increase capture rates so maximized virus detection probability in the mosquitoes can be achieved for assessing risk and outbreak predictions. This can be accomplished by exploiting the host-seeking behavior of adult female mosquitoes and the olfactory cues used to locate a potential host. Here, odors emanating from fur of sheep, a susceptible host for RVFV, is shown to improve trap capture rates of mosquito vectors of RVF in a standard surveillance trap. These data provide for future investigations to identify attractive components present in these natural odors, so that they can be incorporated into existing traps to serve as a population density-monitoring tool for improved arbovirus disease surveillance during IEP.

## Introduction

Rift Valley fever virus (RVFV) is transmitted primarily by mosquitoes and there are periodic outbreaks of this disease in humans and domestic animals in Africa and the Arabian Peninsula [1], [2]. Key mosquito vectors involved in the enzootic transmission include flood water *Aedes* spp. as the primary vectors, and other epizootic culicine vectors such as *Mansonia*, *Culex* and *Anopheles* spp. as the secondary vectors [3]. In Kenya, the number of suspected vectors continues to rise with increasing isolation of the virus from additional species [3]. Since human vaccines and therapeutic treatments are not available for RVFV, surveillance is essential for early warning to ensure that devastating outbreaks and/or sporadic infections are prevented.

Efficient surveillance is essential for early detection of increased vector abundance and detection of pathogens in trapped mosquitoes. This requires a systematic collection of mosquito samples and routine testing of mosquito pools for arboviruses in order to assess the status of transmission and to allow for informed decision-making [4]. However, fluctuations in mosquito abundance and arboviral infections pose a challenge for mosquito based surveillance programs, since different surveillance strategies are required to detect different arboviral vectors and infection rates and potential and transmission rates. This is particularly problematic in the case of early detection and during the inter-epidemic periods (IEP), when transmission foci are sporadic and mosquito infection rates are low. Therefore, detection of mosquito infections when there is low transmission requires the collection of large samples of mosquitoes. For West Nile virus, 700 mosquitoes are needed for a modest detection probability of 0.5 when the natural infection rate is 0.1% for mosquito surveillance programs in the early season or in areas of low transmission [5].

Trapping large numbers of mosquitoes for detection of RVFV can be accomplished by improving the efficiency of existing surveillance traps, such as the standard CO_2_-baited CDC light trap. One way to improve trapping efficiency is by exploiting the host-seeking behavior of female mosquito vectors. Adult female mosquitoes use host-emitted olfactory cues to locate hosts to obtain blood meals [6]. Domestic animals including cattle, sheep, camels and goats serve as hosts for these vectors of RVFV. However, sheep appear to be more susceptible to RVF infections than cattle or camels [7], [8]. Whether or not animal susceptibility is associated with increased attraction is unclear; however, it is clear that sheep are preferred hosts of these vectors. We hypothesized that body odors from sheep are important cues used by RVF mosquitoes. The present study was carried out to investigate the response of mosquito vectors of RVFV to the CO_2_-baited CDC light trap combined with sheep skin odors, in a field setting.

## Materials and Methods

### Study sites

All experiments were conducted at two ecologically distinct sites: Ijara and Marigat districts, which are highly endemic areas for epidemic Rift Valley fever (RVF) in Kenya [3], and are currently under active surveillance for arbovirus activities.

Ijara District is located in the North Eastern Province of Kenya and is characterized by a semi-arid to arid climate. Mosquitoes were sampled at Kotile (1.97°S, 40.19°E) (near Masalani) and Sangailu (1.31°S, 40.71°E), which is around 60 m above sea level. The average annual rainfall is 540 mm with bimodal peaks recorded from March–June and September–December each year. However, the interannual rainfall variability is very high and reaches abnormal levels leading to floods during El Niño years. Minimum temperatures are always above 20°C, and maximum temperatures reach 30°C to 34°C with a high seasonal and interannual variability.

The predominant vegetation is Acacia-Commiphora deciduous bushland and thicket (Savannah, Shrubland, open to very open shrubs), which is much degraded due to overgrazing around the settlement areas. The road leading from Masalani to Sangailu demarcates the boundary between these semi-arid landscapes and the more moist Tana River delta and Boni Forest towards the coast. Boni Forest is an indigenous open canopy forest that forms part of the Northern Zanzibar-Inhamdare Coastal Forest Mosaic.

The second study site is Marigat district, located in the Kenyan Rift Valley 250 km northwest of Nairobi where traps were set in surrounding villages/communities of N'gambo (0.50°N, 36.06°E) and Salabani (0.55°N, 36.06°E). The study site covers the basin between Lake Baringo and Lake Bogoria with the town of Marigat as an economic center and lies about 1000 m above sea level. The climate is hot and dry with high rainfall variability, both annually and inter-annually. The average annual rainfall is 650 mm with weak bimodal peaks recorded from March–May and June–August. Temperatures vary from 30 to 35°C, but can rise to 37°C in some months.

The low lying arid part of the Baringo basin consists of northern Acacia-Commiphora bushlands and thickets but it has experienced severe land degradation caused by uncontrolled grazing and deforestation. *Prosopis juliflora* (Sw.) DC, locally called mathenge, was introduced to Baringo in the early 1980s for fuelwood production and reforestation as a mitigation measure to stop desertification. The plant was introduced at two sites but now covers large areas, i.e. N'gambo village, one of the vector sampling sites.

Three indigenous human communities live in this area, the Ilchamus, Pokot and Tugen. They earn their living through pastoralism and agro-pastoralism keeping large numbers of cattle and small livestock such as sheep and goats. The Perkerra irrigation scheme (growing of vegetables, maize seed production), fishing and tourism provide additional income to these communities.

### Choice of animal

Sheep are the most susceptible among livestock hosts afflicted by RVFV [1], [8], [11], and the living animal has been exploited as a lure in trapping mosquito vectors [12]. Its role in the enzootic maintenance of the RVFV [13] is the reason why it is the preferred domestic animal currently being used as sentinels in an ongoing surveillance program for RVF at the two study sites.

### Ethics statement

The study was conducted with the approval of the national ethics review committee based at the Kenya Medical Research Institute (KEMRI) and is renewed on an annual basis after a scientific audit. The Animal use component was also given approval by the KEMRI Animal Use and Care committee (KEMRI-AUCC). KEMRI-AUCC complies with the national guidelines for care and use of laboratory animals in Kenya developed by the Kenya Veterinary Association and the Kenya lab animal technicians association 1989. The KEMRI-AUCC which approved the study protocol has an assurance identification number A5879-01 from the Office of Laboratory Animal Welfare (OLAW) under the Kenyan department of health and human services. For purposes of livestock use, funds from the project were used to purchase animals to monitor RVFV seroprevalence and used for all experimental activities described in this study. These animals were owned and maintained for the study by the project. The project bought 492 animals comprising 5 sentinel herds; two in Marigat, three in Ijara district (one in Kotile and 2 in Sangailu). The animals were left with the owners as part of their flocks but they were not allowed to sell or slaughter them because the project was monitoring the animals. The animals were reverted back to the owner at the end of the project activity. Any newborns born out of the tagged animals belonged to the farmers. We worked in collaboration with the department of veterinary services and veterinary doctors mandated by the government to do livestock sampling and research. The above terms were stipulated well in an agreement between the farmers and the International Centre of Insect Physiology and Ecology (*icipe*), the hosting institution for the AVID Project Consortium.

### Experimental design

Experiments were conducted in October and December 2010 during the rains to ascertain the presence of mosquitoes. This comprised 10 replicates of 4 treatments per district. The treatment-trap combinations consisted of the standard CDC light trap alone (L) and baited with (a) animal volatiles (LF), (b) CO_2_ (LC), or (c) CO_2_ and animal skin volatiles (LCF) using fur obtained from living sheep. The animal volatiles consisted of fresh sheep (*Ovis aries* Linnæus) hair samples shaved from the belly and back areas of the animals (avoiding the head and anal regions) daily. The animal fur was wrapped in five layers of aluminum foil, kept in a cold box (10°C) and immediately transported to the trapping site (located between 2 to 5 km). Once at the trapping site, approximately 19 g of the animal fur were placed in each canister (cylindrical in shape with a diameter of 9.5 cm and height 22.5 cm) designed from Brass mesh wire (mesh size, 0.15 mm, McNichols Co, Tampa FLA). With an inter-trap distance of 40±2 m, the traps were hung in trees 1.5±0.2 m off the ground and activated within 30 min of sunset (1800–1830) and trap contents collected within 30 min after sunrise (0600–0630 hours). Treatments and control were assigned to a predetermined similar area following a Latin square design with days as replicates. Traps were rotated on every trapping day to minimize variability due to trap placement. Dry ice (1 kg) was used as the CO_2_ source, which was delivered in Igloo thermos containers (∼2 L) (J.W. Hock, Gainesville, FL) with a 13-mm hole in the bottom center. Treatments with the canisters containing fur (which released skin volatiles) were hung at the base of the standard CDC trap (battery-powered model 512, John W Hock Co., Gainesville, FL) and when in the presence of CO_2_ directly in the air flow. All bait canisters were boiled in l0% bleach solution after each nightly trapping to eliminate any residual odor.

### Mosquito processing

Mosquitoes caught daily from each of the treatments were anesthetized using triethylamine and identified morphologically to species using taxonomic keys 14–16. When large numbers of mosquitoes were trapped, they were anesthetized, sorted from other insects and immediately stored in 15 or 50 mL centrifuge tubes, and transported in a liquid nitrogen shipper to the laboratory where they were later identified and the total number by species for each treatment-trap were recorded.

### Data analyses

Trap count data were analyzed per district and were also subdivided into four categories (i.e., key primary vectors, primary vectors, secondary vectors and non-vectors) based on the relative importance and involvement of member species in RVFV transmission 2,3. The four main categories of trapped mosquitoes recorded in the different treatments were further categorized as follows: flood water *Aedes* species (key primary vectors; *Aedes mcintoshi* and *Aedes ochraceus*); primary vectors (*Aedes sudanensis*/*Aedes tricholabis*); secondary vectors (*Mansonia* and *Culex* spp.) and non-vectors, which do not fall into any of these categories (Table 1). Analysis of key primary and primary RVFV vectors was limited to Ijara district where they were mainly encountered and secondary vectors limited to Marigat district where they occurred in substantial numbers (Table 1). Daily count of mosquitoes recorded in the various trap treatments were analyzed using a generalized linear model with negative binomial error structure and log link using R 2.11.0 software [17]. Using the treatment L only (control) as the reference category, the incidence rate ratios (IRR) that mosquito species chose other treatments (LCF, LC and LF), instead of the control, were estimated. The IRR for the control is 1 (unity) and values above this indicates better performance and values below under performance of the treatments relative to the control.

**Table 1:** Number of each mosquito species captured by baited and unbaited CDC light traps at two districts in Kenya.

	Marigat district				Ijara district			
RVFV vector group	L	LC	LCF	LF	L	LC	LCF	LF
**Key primary vectors**								
*Ae. mcintoshi*	5	7	8	3	20	141	↑208	27
*Ae. ochraceus*	0	0	0	0	470	856	↑1034	648
**Primary vectors**								
*Ae. sudanensis*	0	0	0	0	4	18	30	4
*Ae. tricholabis*	0	0	0	0	85	2,794	↑3745	251
**Secondary vectors**								
*Culex poicilipes*	445	5,154	↓3522	696	1	9	24	3
*Cx. ethiopicus*	0	5	7	1	0	0	1	1
*Cx. bitaenorrhynchus*	5	14	38	2	0	0	0	0
*Cx. pipiens*	49	457	↑657	53	7	53	34	10
*Cx. tigripes*	0	4	4	2	0	0	0	0
*Cx. univittatus*	30	181	234	37	6	14	9	12
*Cx. vansomereni*	0	6	2	0	0	0	0	0
*Mansonia africana*	6,223	17,521	↑24254	6,484	0	2	0	0
*Ma. uniformis*	2,124	5,563	↑7334	1,912	1	0	2	0
**Non-vectors**								
*Aedes furcifer*	0	0	0	0	0	1	2	0
*Ae. hirsutus*	0	3	2	1	0	0	0	0
*Ae. metallicus*	0	0	0	0	1	2	2	0
*Ficalbia splendens*	376	368	354	265	0	0	0	0
*Aedomyia furfurea*	0	0	0	0	0	4	5	0
*An. coustani*	744	1,324	↓1315	826	1	3	2	1
*An. funestus*	0	1	1	2	0	0	0	0
*An. pharoensis*	27	18	51	20	0	0	0	0
*An. squamosus*	0	9	5	1	1	3	3	2
*An. gambiae* s.l.	20	45	66	46	0	0	0	0
*Coquilettidia aurites*	0	2	6	0	0	0	0	0
*Cq. metallicus*	0	1	1	0	0	0	0	0

L, light only; LF, light+animal odor; LC, light+CO_2_; LCF, light+CO_2_+animal odor; ↑, increase in captures in LCF traps relative to LC; ↓, decrease in captures in LCF traps relative to LC.

## Results

### Species abundance and composition

**Figure 1:**
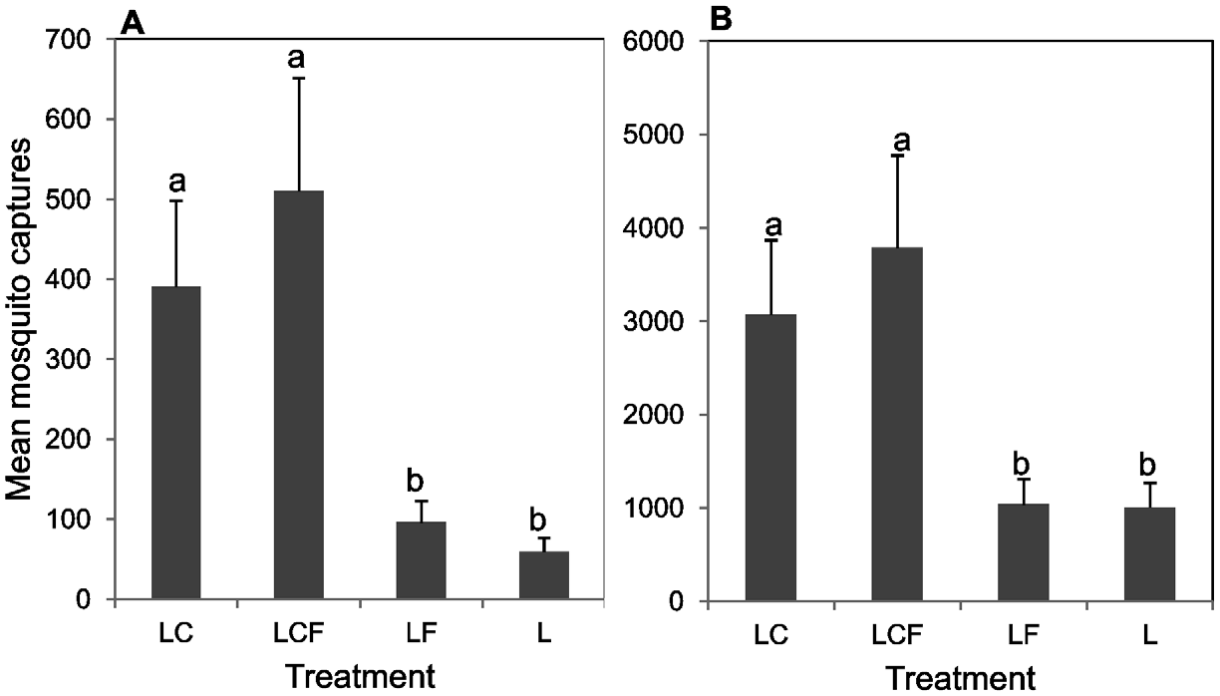
Mean mosquito captures in 10 replicate trials per treatment at the two districts in Kenya. A) Ijara district; B) Marigat district. Bars followed by similar letters are not significantly different at P = 0.05. L, light only; LF, light+sheep odor; LC, light+CO_2_; LCF, light+CO_2_+sheep odor.

**Figure 2:**
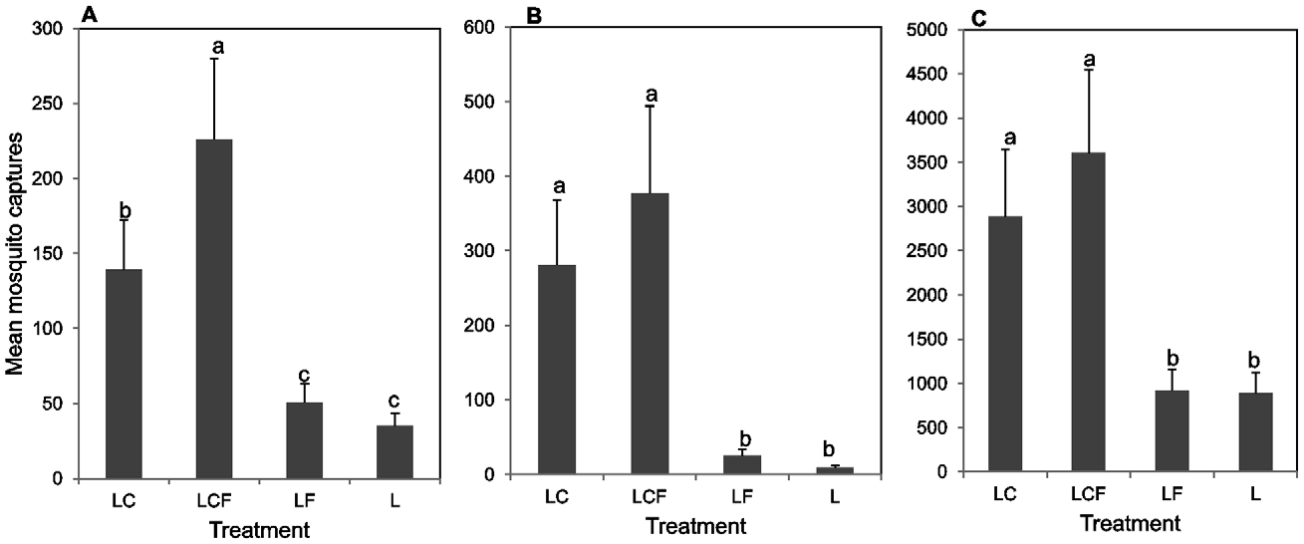
Mean mosquito captures/trap/night for different RVFV vector groups in 10 replicate trials/district in Kenya. A) Key primary vectors; B) Primary vectors; C) Secondary vectors. Bars followed by similar letters are not significantly different at P = 0.05. L, light only; LF, light+sheep odor; LC, light+CO_2_; LCF, light+CO_2_+sheep odor.

The distribution of RVFV mosquitoes captured per treatment-trap combination for the two districts are contained in Table 1. Mosquito species composition and trap captures differed markedly between the two districts which might suggest varied habitat preferences for each mosquito species. Differences in abundance were observed between the treatments with no clear pattern of preference of any species for a particular trap treatment. Some species were not caught in all replicates, and it is unclear if such variability was due to overall low population densities or the mosquitoes failing to enter (or to respond to) the traps. In general, traps baited with CO_2_ (LCF and LC) captured more mosquitoes than those without (LF and L) (Table 1 and Figures 1 and 2).

### Effect of treatment on overall mosquito captures

There was a significant effect of treatments compared to the unbaited CDC trap on overall mosquito captures from Marigat (χ^2^ = 20.68, df = 3, p<0.001) and from Ijara (χ^2^ = 37.51, df = 3, p<0.001). Trap catches from Marigat indicate that, compared to L only, LC and LCF traps caught 3–4 times more host-seeking mosquitoes [IRR = 3.1 for LC and IRR = 3.8 for LCF]. LCF traps recorded higher mosquito catches compared to LC traps (IRR = 1.23) although the difference was not statistically significant (Figure 1). Similarly, the LF trap caught slightly more mosquitoes (IRR = 1.03) than L only but was not significantly different (Figure 1). Similar findings were observed at Ijara where there was a significant treatment effect on mosquito catches (χ^2^ = 37.51, df = 3, p<0.001). Carbon dioxide (LC), CO_2_+fur (LCF) significantly increased trap captures by 6.5 and 8.5 times, respectively, compared to the control. The LF caught more than the control, L, but this was not statistically significant (Figure 1).

### Treatment effect per vector category per district

Trap catches at Ijara were dominated by flood water aedine mosquitoes categorized as key and primary RVFV vectors; these species were sparse or absent at Marigat. There was a highly significant effect of treatments on key primary RVFV vectors (χ^2^ = 199.99, df = 3, p<0.001). For this group, relative to the control, there was a 4.0- and 6.5-fold significant increase in captures recorded in LC and LCF traps, respectively (Figure 2). Additionally, LCF capture rates were significantly higher than LC capture rates (IRR = 1.63).

A significant effect of treatments on RVFV primary vectors was also evident (χ^2^ = 74.24, df = 3, p<0.001). Compared to the control, the treatments LF, LC and LCF caught 2.9, 31 and 42 times as many primary vectors. Interestingly, for this group, there was a 34% significant increase in captures for traps baited with LCF compared to LC (IRR = 1.34) (Figure 2).

Marigat yielded very low catches for key primary vectors and there was a total absence of primary vectors. Therefore results are only presented for secondary vectors. For secondary vectors at Marigat, there was a highly significant effect of treatments on the mosquito catches (χ^2^ = 22.94, df = 3, p<0.001). Relative to the control, there were 3 to 4-fold increases in captures for LC and LCF traps, respectively (Figure 2). Comparable captures were recorded for LF and L traps with only a slight increase recorded in LF baited traps relative to the control (IRR = 1.04). Captures rates, although not significant were higher for LCF traps than LC traps (IRR = 1.24) (Figure 2). Mosquito collections within this category at Ijara were low and dominated by *Cx. pipiens* s.l. with an observed increase in captures in the other treatments compared to L.

The non-vectors category included species of the genera *Ficalbia*, *Coquilettidia*, *Anopheles* and *Aedes* (Stegomyia). Members of these genera occurred in low numbers in both districts, especially at Ijara (Table 1). However, data for Marigat suggest a bias in trap captures in LCF and LC, compared to L although there were no significant differences in the captures between these treatments, while similar trap captures were observed for the LF and L-baited traps. Non-mosquito species notably beetles and moths were trapped in addition to mosquitoes but were not included in our data.

## Discussion

### Effect of sheep fur on trap captures

The results demonstrate that more mosquitoes were caught in traps that contained a release of the combination of sheep odors+CO_2_ and were in most cases the most attractive bait compared to the conventional CO_2_-baited light trap. This confirms that odors emanating from sheep fur play a role in host-location by these mosquitoes. The attractive effect was highly evident in captures of flood water aedines comprising key and primary RVFV vectors as well as secondary vectors. The effectiveness of sheep is supported by a study on blood meal patterns during a RVF outbreak where widespread feeding on sheep was observed [18]. Moreover, most mosquitoes belonging to the *Culex*, *Mansonia* and *Aedes* genera have been reported to feed opportunistically and readily on mammals [19]–[21].

The entire animal body emanations comprising breath and skin volatiles influence the outcome of mosquito host-seeking process [22]. Research has indicated that animal skin emanations have a kairomonal (attractive) effect on mosquitoes while breath volatiles have an allomonal or repellent effect [23]. Skin body odor may be the primary factor for mosquito attraction and discrimination when mosquitoes are in close proximity of a host. It is therefore not surprising that addition of skin volatiles captured in sheep fur enhanced captures of mosquitoes attracted to sheep hosts when combined with the conventional CO_2_-baited light trap.

The effect of sheep skin odors emanating from fur was not evaluated alone but in combination with CO_2_ and/or light which are known attractants for mosquitoes and other biting flies. Although animal odors enhanced trap captures when added to either CO_2_-baited light trap or light trap only, the captures were greater in the CO_2_-based blend than in the combination without CO_2_. However, the crude animal skin odor in traps is imperfect because of possible loss of volatile attractive components over time compared to the dynamic production from live animals. Therefore it would be beneficial to identify the attractive compounds and develop a synthetic blend.

In some replicates there was a decrease in trap catches when host odor was added to light or to CO_2_. This could be attributed to variation in attractiveness of the batches of animal fur used in the daily trapping experiment as odors used were not from the same animal; low occurrence of targeted mosquitoes, as observed at the districts for certain vector categories; a difference in preferred host other than sheep e.g. *Cx. poicilipes* between LCF and LC baited traps (Table 1); and volatiles from fur are a static system and most volatile compounds evaporate first and therefore the odor profile changes. The effect of host odors did not markedly influence trap catches of the non-vector category. The low abundance of mosquitoes was insufficient to observe a significant preference in trap catches for the different treatments used; even though there was an increase in trap catches for those baited with host odors compared to light only.

The effect of CO_2_ on trap catches was not evaluated independently; however, its effect was evidenced in the difference in trap catches between LC and L baited traps. The data support its role in enhancing trap captures [24], especially for RVFV vectors. Our experimental setup excluded landing response as a measurement, instead focussing solely on trap catch. The goal was to evaluate animal fur containing skin emanations that provided attractive stimuli. However, the large response of these mosquitoes to CO_2_, suggests that it can serve as a good positive control for evaluating candidate synthetic attractants of skin origin for this group of arbovirus vectors of medical and veterinary importance.

Our preliminary trials (data not shown) and earlier studies highlighted the importance of these attractants in flight activation of mosquitoes towards host odors [25], [26]. This justifies the inclusion of these well-known long-range attractants in trap design. Our data suggest that host skin odors other than CO_2_ are important in enhancing mosquito trap captures in concurrence with studies reporting enhanced effect of mosquito attraction to animal skin volatiles in the presence of CO_2_ or light [22], [27], [28].

### Role of carbon dioxide (CO_2_) and light

It is well-known that many nocturnally-active hematophagous insects are attracted to light [29], [30]. In conformity with earlier findings [31], our results show that light as a visual cue is enhanced by sheep skin odors and CO_2_. Besides being non-specific, previous studies have argued that CO_2_ activates mosquitoes to initiate host-finding, but may not necessarily attract it and at close range, can actually act as a deterrent [26] and be of limited use in host discrimination [32]. Although this was not the subject of our study, CO_2_ increased trap captures in the presence of host skin odors, in agreement with previous research [24], [25], [33].

The observed trap captures recorded in the LCF traps were generally higher compared to those caught in the LC traps. Nonetheless, among the mosquito species trapped, differences in capture rate were not observed between the LCF and LC traps. Therefore, CO_2_-baited light traps may be adequate for monitoring and surveillance of these species. However, for effective arbovirus disease surveillance, an improved sampling method is vital especially during the inter-epidemic period where transmission foci are sporadic and infected vectors are rare. Emphasis needs to be placed on increasing the collections with an additional advantage of depicting the dynamics of populations.

### Less attractive, unattractive or just different?

Beyond the already described finding that animal odor inclusion increases trap catch with CO_2_ present, there were some cases where it suppressed trap catch. In some cases, lower catches of the LCF trap were noted on days with light showers; therefore, precipitation may have interfered and reduced mosquito attraction to skin odor baits as observed before by Olanga *et al.* [34]. However there was no record of variation in weather patterns during the study period. Another possibility is the variation from fur samples used in this study. Samples were obtained from various animals without prior assessments of their degree of attractiveness. Animals in a herd are known to vary greatly in their attractiveness to mosquitoes [35], [36]. Reduced attraction due to loss of important volatile compounds during the fur extraction process remains plausible.

A higher number of *Cx. poicilipes* were collected in CO_2_-baited light traps than in similar traps baited only with host skin odor, although the difference in trap captures was not significant. This suggests that sheep are not preferred hosts for this species. However, the effect of CO_2_ in the presence of host-related odors may be variable and a strong attraction response may be observed with often different responses between species [37], [38]. This observation might emphasize the importance of trap placement in the sampling process, though it is not certain if this species would be attracted to the host that emanates the greatest amount of CO_2_ in nature.

Differential catches of *Cx. pipiens* s.l. and *An. gambiae* s.l. to odors from sheep fur were recorded at the different sites (Table 1). Among the species complexes captured, there are known marked differences in olfactory responses between members of the complexes [39], [40]. *Culex pipiens* preferentially feed on birds [41] although they can adapt and readily feed on mammals in proportions possibly based on host abundance [42]. The observed differences in trap responses in the highest trap treatments (i.e., LC and LCF) at both districts may indicate different spatial feeding preferences in geographically separate populations. Related response patterns of discrete populations of mosquito species to host odors has been reported [42], [43], as such, it may be worthwhile to include a preference test involving odors from other livestock hosts in field bioassays.

Only volatiles from skin emanations captured in the fur were tested in this study. Studies on other volatile sources involved in host attraction to hematophagous flies have been reported from feces [44] and urine [45], [46]. In this regard, other sources of attractive odors might contribute to the attraction of mosquitoes and combination using these odors may be worth investigating.

Laboratory bioassays have commonly been used to evaluate the effect of semiochemicals on mosquito behavior whilst minimizing other environmental variables. However, such an approach is inadequate for predicting effects on natural populations and on ecosystem-level features [47]. Alternatively, insect behaviors have been assessed in the field by baiting traps with extracts of animal volatiles [42], [48]. Use of whole animals provides another approach but it becomes difficult to delineate individual contributions of attractants from breath or skin emanations or other exogenous compounds to the overall trap catches. Our design followed a field-based approach to evaluate the role of skin emanations on mosquito trap catches. The design can account and provide for an understanding of heterogeneities which dramatically influence dynamics of natural systems. This is similar to the trapping design employed by Njiru *et al.* [49] and Jawara *et al.* [50] to investigate mosquito captures in conventional traps baited with human foot odors trapped on nylon stockings.

Although the contribution of geographical variability to the total variance was not estimated, possible experimental confounders such as time, location and environmental influence are unlikely to affect the overall observed results as the present experiments were performed at a variety of sites with different animals of the same species and treatment traps treated alike. As such, it is likely that many of the mosquitoes approaching the trap had the opportunity to sample more than one of the treatment-traps, and may have made a choice between them. Albeit the above mentioned challenges, the use of crude volatiles in the field approach presented in this paper can contribute to the evaluation of the effect of host volatiles in the standard CDC light trap.

In conclusion, the addition of sheep skin odor to the CO_2_-baited light trap improved trap catches of RVFV vectors in line with similar findings reporting enhanced effect of animal skin odors and other cues such as CO_2_ [27], [28], [44]. Our results indicate host skin olfactory cues are important signals in mediating mosquito host location. The finding is also in accordance with the consensus that additional compounds other than CO_2_ from animal skin may be exploited by mosquitoes in host location [51]–[53]. Sheep skin odor contributes to the attraction of host-seeking RVFV mosquito vectors. Identification of chemicals emanated by sheep might provide the basis for the development of improved devices to sample these vectors. However, refinements into an effective monitoring tool requires identifying and understanding the specific behavioral effects of the attractive components present in these skin odors which is currently underway.
